# How substandard dwellings and housing affordability problems are associated with poor health in a vulnerable population during the economic recession of the late 2000s

**DOI:** 10.1186/s12939-015-0238-z

**Published:** 2015-11-04

**Authors:** Ana M. Novoa, Julia Ward, Davide Malmusi, Fernando Díaz, Mercè Darnell, Carme Trilla, Jordi Bosch, Carme Borrell

**Affiliations:** Agència de Salut Pública de Barcelona, Barcelona, Spain; Institut d’Investigació Biomèdica (IIB Sant Pau), Barcelona, Spain; Gillings School of Global Public Health, University of North Carolina, Chapel Hill, USA; CIBER de Epidemiología y Salud Pública (CIBERESP), Barcelona, Spain; Cáritas Diocesana de Barcelona, Barcelona, Spain; Universitat Pompeu Fabra, Barcelona, Spain

**Keywords:** Perceived health, Mental health, Housing affordability, Economic recession

## Abstract

**Introduction:**

Given the increasing number of people in Spain struggling to pay housing-related costs during the economic recession, it is important to assess the health status of these communities as compared to the general population and to better understand the different housing dimensions that are related with poor mental health. This study aims to describe the housing conditions and health status of a sample of people assisted by Caritas Barcelona (Spain) and living in inadequate housing and/or struggling to pay their rent or mortgage, to compare the health outcomes of this population with those of the overall population of Barcelona, and to analyze the association between housing dimensions and mental health.

**Methods:**

We used a cross-sectional design. The participating adults (*n* = 320) and children (*n* = 177) were those living in the dioceses of Barcelona, Sant Feliu and Terrassa (Spain) in 2012 and assisted by Cáritas. They were asked to answer to three questionnaires on housing and health conditions. Eight health related variables were used to compare participants with Barcelona’s residents and associations between housing conditions and poor mental health were examined with multivariate logistic regression models.

**Results:**

In Barcelona, people seeking Caritas’s help and facing serious housing problems had a much poorer health status than the general population, even when compared to those belonging to the most deprived social classes. For example, 69.4 % of adult participants had poor mental health compared to 11.5 % male and 15.2 % female Barcelona residents. Moreover, housing conditions were associated with poor mental health.

**Conclusions:**

This study has shown how, in a country hit by the financial recession, those people facing housing problems have much worse health compared to the general population.

## Introduction

Housing conditions impact health through a variety of interrelated mechanisms including the emotional link with the dwelling, housing affordability, physical dwelling conditions, and the physical and social (community) environment of the neighbourhood where the dwelling is located [1, 2]. Failings in any of these dimensions of housing can negatively impact the health of a home’s occupants.

Previous studies, mainly undertaken in the USA, have found associations between housing instability (which implies having multiple ongoing difficulties, both personal and economic, associated with maintaining the residence) and wellbeing/mental health [3–10]. Qualitative studies have mainly focused on exploring how the process of housing affordability and the process of eviction leads to poor health and wellbeing [11–13]. Examples of these mechanisms include uncertainty, fear and lack of control; the impact on social status and sense of self; stigma, shame and embarrassment, failure and a loss of emotional capital [11]. Other studies have described how individuals with housing instability were more likely to have self-declared hypertension and heart disease [6, 14]. Moreover, unhealthy behaviours may be adopted to cope with the stress of problems with housing affordability, such as tobacco use, alcohol consumption, or unhealthy eating, which are related to subsequent physical health outcomes, such as cardiovascular disease [6, 11, 15].

Spain is one of the European countries where the consequences of the global economic crisis of the late 2000s are being more evident [16] and where the distinctive features of its housing system have exacerbated the consequences of the crisis. Historically, housing in Spain has been used as a speculative good instead of a first necessity good, making access to adequate housing difficult for low-income families. While Spain is the OECD country with the largest housing stock per inhabitant, it is highly underutilized (14 % unoccupied and 16 % used as second home) despite its increasing homeless population. In addition, social housing only represents between 1 % and 2 % of housing stock (one of the lowest in the European Union-EU-), and budget for rental assistance programmes is also among the lowest in the EU) [1, 17, 18]. The impact of the economic crisis on access to adequate housing in Spain has been mainly due to the reduction of household income, leading to difficulties in paying housing-related costs and, potentially, to the loss of the dwelling. Foreclosure procedures increased from 58,000 in 2008 to 101,034 in 2012 and the number of homeless population in Barcelona increased by 43 % between 2008 and 2013 [19].

Given the scarcity of literature in Spain analyzing the association between housing and health and given the increasing number of people in Spain struggling to pay housing-related costs and/or living in substandard housing, it is important to assess the health status of these communities as compared to the general population and to better understand the different housing dimensions that are related to poor mental health.

Caritas is one of the institutions focused on helping people with housing problems. It is an institution linked to the Catholic Church whose main focus is on direct care for socially vulnerable people. One of its services is to arbitrate between families and owners or financial institutions that issued the loan in order to find solutions and avoid foreclosure. It also provides financial assistance to families, and in some cases, relocation into homes owned by Caritas.

This study aims to describe the housing conditions and health status of a sample of people assisted by Caritas Barcelona (Spain) and living in inadequate housing and/or with difficulties in paying their rent or mortgage, to compare the health outcomes of this population with those of the overall population of Barcelona, and to analyze the association between housing dimensions and mental health.

## Methods

### Design, data sources and study population

This study uses a cross-sectional design. The participating families were those living in the dioceses of Barcelona, Sant Feliu and Terrassa (Catalonia, Spain) in 2012 and assisted by Caritas. They were identified from the Caritas users’ registry. Participants were asked to answer to three questionnaires, delivered by a trained interviewer who conducted the face-to-face interview at the Caritas’s centre in Barcelona with a randomly selected adult member of each family. The questionnaires consisted of: (1) a housing questionnaire, which collected information on household composition, housing type and habitability, housing affordability, and satisfaction with housing and neighbourhood conditions; (2) a resident questionnaire, which gathered information on several health indicators as well as socio-demographic characteristics; and (3) a child questionnaire, which included health and socio-demographic information for a randomly selected child aged 4 to 14 years old residing in the household, reported by the selected adult member.

In addition, comparison with the general population was performed using the Barcelona Health Survey of 2011, based on face-to-face interviews in the homes of a representative sample of the non-institutionalised Barcelona residents.

The study population consisted of 320 adults: 175 assisted by Caritas’s Direct Assistance Service (DAS), living in substandard and/or overcrowded housing and considered to be a priority for rehousing, and 145 assisted by Caritas’s Housing Mediation Service (HMS), at risk for being evicted from their owned or rented housing due to economic problems. Information was also obtained for 177 children, 93 (54.8 %) belonging to families assisted by Caritas’s DAS. The overall response rate was 45 %.

The study was approved by the Ethics Committee of the Parc de Salut Mar (Barcelona).

### Study variables

The outcome variables included several health indicators. In adults: fair or poor self-reported health; below the study population median quality of life score measured using the EuroQol questionnaire ED-5 [20]; poor mental health (score of more than 2 in the Goldberg's General Health Questionnaire 12 items, assessing symptoms of anxiety and depression) [21]; self-reported depression or anxiety the previous year; use of tranquilizers, antidepressants, or sleeping pills the previous 2 days; self-reported migraine or frequent headaches during the previous year; self-reported backache during the previous year; and averaging less than 6 hours of sleep per night during working days. In children: poor or fair general health status; poor mental health measured using the Strengths and Difficulties questionnaire (SDQ; "abnormal" (>16) score of the total difficulties scale) [22]; recurrent otitis during the previous year; and not having breakfast every day before leaving home.

Several socio-demographic and housing characteristics were included as explanatory variables (see variables in Table 1). Social class was obtained through current or past occupation of the adult interviewed using the Spanish classification of the Spanish Society of Epidemiology [23]. Social support was measured by means of the Duke Scale considering scores below 32 as inadequate support.

**Table 1:** Socio-demographic characteristics of the population assisted by Caritas Barcelona

	Direct Assistance Services		Housing Mediation Service	
	Men	Women	Men	Women
	(*n* = 45)	(*n* = 130)	(*n* = 72)	(*n* = 73)
	n (%)	n (%)	n (%)	n (%)
**Socio-demographic characteristics**				
Age (years)				
16–29	5 (11.1)	33 (25.6)	2 (2.8)	4 (5.5)
30–44	27 (60.0)	81 (62.8)	35 (48.6)	40 (54.8)
45–64	12 (26.7)	15 (11.6)	34 (47.2)	23 (31.5)
≥ 65	1 (2.2)	0 (0.0)	1 (1.4)	6 (8.2)
Social Class				
I, II, III (non-manual)	1 (2.3)	10 (8.3)	10 (13.9)	12 (17.1)
IV (skilled and semi-skilled manual)	25 (58.1)	57 (47.5)	49 (68.0)	36 (51.5)
V (unskilled manual)	17 (39.6)	53 (44.2)	13 (18.1)	22 (31.4)
Education degree				
Primary or less	15(33.3)	37 (28.5)	20 (37.8)	21 (28.8)
Secondary	24 (53.4)	74 (56.9)	40 (55.5)	42 (57.5)
Bachelor’s degree or more	6 (13.3)	19 (14.6)	12 (16.7)	10 (13.7)
Foreign-born	40 (88.9)	124 (95.4)	44 (61.1)	40 (54.8)
Legal status				
Spanish or member of the EU	8 (17.8)	19 (14.7)	41 (56.9)	46 (63.0)
Legal immigrant	20 (44.4)	51 (39.6)	30 (41.7)	25 (34.3)
Undocumented immigrant	17 (37.8)	59 (45.7)	1 (1.4)	2 (2.7)
Employment status				
Employed	11 (24.4)	69 (53.1)	10 (13.9)	32 (43.8)
Unemployed	30 (66.7)	53 (40.7)	55 (76.4)	32 (43.8)
Other situation^a^	4 (8.9)	8 (6.2)	7 (9.7)	9 (12.4)
Family composition				
Couple and children	25 (55.5)	49 (37.7)	47 (65.3)	17 (23.3)
Single parent	3 (6.7)	53 (40.7)	2 (2.8)	25 (34.2)
Alone	5 (11.1)	5 (3.9)	8 (11.1)	15 (20.6)
Other composition	12 (26.7)	23 (17.7)	15 (20.8)	16 (21.9)
Social support				
Adequate	16 (36.4)	42 (32.8)	21 (29.6)	17 (23.9)
Inadequate	28 (63.6)	86 (67.2)	50 (70.4)	54 (76.1)

^a^Student, housekeeper, permanent disability leave, retired

The housing variables included different aspects of the following 4 dimensions: affordability, emotional link with the dwelling, dwelling conditions and neighbourhood and community conditions (see variables in Table 2) [1]. To measure the participant’s emotional link to the dwelling, a composite score (Cronbach’s Alpha of 0.82) was created by averaging the 5-point Likert-scale responses to questions regarding the resident’s overall satisfaction with the dwelling, satisfaction with the dwelling’s space, satisfaction with the dwelling’s views, the belief that others would be happy with this dwelling, the sense of security felt in the dwelling, and whether the dwelling expresses his/her personality and values. The number of habitability problems included not having any type of ventilation in one or more of the bedrooms or the dinning room, having often or always dampness and mould, cold or heat being always identified as a problem during winter or summer, respectively, having had a serious infestation (rats, mice, cockroaches, fleas, bedbugs) during the previous year, and insufficient amount of daylight (having to switch on the lights during the day) (Cronbach’s Alpha of 0.48). The number of housing-related material deprivations included central heating, air conditioned, dishwasher, computer, and Internet connection (Cronbach’s Alpha of 0.63) [25] Overcrowding was defined as more than one person per room, excluding toilets and including members of other families.

**Table 2:** Housing characteristics of the population assisted by Caritas Barcelona

	Direct Assistance Services		Housing Mediation Service	
	Men	Women	Men	Women
	(*n* = 45)	(*n* = 130)	(*n* = 72)	(*n* = 73)
	n (%)	n (%)	n (%)	n (%)
**Affordability**				
% of monthly household income spent on rent or mortgage				
< 30 %	3 (6.8)	18 (14.9)	6 (9.1)	10 (14.7)
30–50 %	16 (36.4)	43 (35.5)	22 (33.3)	16 (23.5)
> 50 %	25 (56.8)	60 (49.6)	38 (57.6)	42 (61.8)
Problems meeting monthly costs				
Strongly disagree, disagree, indifferent	3 (6.7)	22 (17.7)	7 (10.0)	9 (12.7)
Agree	14 (31.1)	34 (27.4)	16 (22.9)	17 (23.9)
Strongly agree	28 (62.2)	68 (54.9)	47 (67.1)	45 (63.4)
In the last 12 months, worried about not having sufficient money for food				
No	7 (15.6)	30 (23.3)	17 (23.6)	17 (21.9)
Yes	38 (84.4)	99 (76.7)	55 (76.4)	57 (78.1)
Time in current house				
< 2 years	25 (55.6)	68 (52.3)	23 (31.9)	30 (41.1)
≥ 2 years	20 (44.4)	62 (47.7)	49 (68.1)	43 (58.9)
Has ever lived in a shack, shelter, car, or on the street				
No	33 (73.3)	107 (82.3)	54 (75.0)	58 (79.4)
Yes	12 (26.7)	23 (17.7)	18 (25.0)	15 (20.6)
**Emotional link with the dwelling**				
Very unsatisfied or unsatisfied	16 (35.6)	38 (29.2)	16 (22.2)	24 (32.9)
Indifferent	13 (28.8)	35 (26.9)	24 (33.3)	22 (30.1)
Very satisfied or satisfied	16 (35.6)	57 (43.9)	32 (44.5)	27 (37.0)
**Dwelling Conditions**				
Housing type				
Apartment/single family home (rented)	23 (51.1)	56 (43.1)	25 (34.7)	32 (43.8)
Apartment/single family home (owned)	1 (2.2)	2 (1.5)	37 (51.4)	25 (34.3)
Room (part of a house)	14 (31.3)	52 (40.0)	4 (5.6)	6 (8.2)
Other^a^	7 (15.6)	20 (15.4)	6 (8.3)	10 (13.7)
Number of habitability problems				
0	4 (9.8)	16 (13.2)	22 (31.9)	13 (18.6)
1	13 (31.6)	38 (31.4)	18 (26.1)	23 (32.9)
2	12 (29.3)	30 (24.8)	17 (24.6)	21 (30.0)
3–5	12 (29.3)	37 (30.6)	12 (17.4)	13 (18.6)
Number of housing-related material deprivations				
0–2	7 (15.6)	22 (17.5)	28 (38.9)	17 (23.3)
3	13 (28.9)	34 (27.0)	17 (23.6)	21 (28.8)
4	9 (20.0)	27 (21.4)	13 (18.1)	24 (32.8)
5	16 (35.5)	43 (34.1)	14 (19.4)	11 (15.1)
Overcrowding^b^				
≤ 1 ppr	18 (40.0)	57 (44.5)	56 (77.8)	57 (78.1)
> 1 ppr	27 (60.0)	71 (55.5)	16 (22.2)	16 (21.9)
**Neighbourhood and community conditions**				
Evaluation of the neighbourhood as a place to live				
Very good, good or indifferent	37 (82.2)	107 (82.3)	59 (81.9)	55 (75.3)
Very bad or bad	8 (17.8)	23 (17.7)	13 (18.1)	18 (24.7)
Noise				
Noise does not impede sleep	30 (66.7)	76 (58.9)	43 (60.6)	31 (42.5)
Noise does impede sleep	15 (33.3)	53 (41.1)	28 (39.4)	42 (57.5)
Safe neighbourhood (when returning home at night) where violence is not a problem				
Strongly agree or agree	25 (55.6)	68 (53.1)	42 (58.3)	33 (46.5)
Strongly disagree or disagree or indifferent	20 (44.4)	60 (46.9)	30 (41.7)	38 (53.5)

*ppr* persons per room

^a^Guesthouse, bed in shared bedroom, shelter, friend’s or family’s house, among others; ^b^More than one person per room, excluding toilets and including members of other families

## Statistical Analysis

Descriptive and bivariate analyses were carried out for the outcome, explanatory, and adjusting variables. Outcome variables were compared with the overall population of Barcelona, as well as those from the most deprived social classes of the city (classes IV and V, which correspond to manual workers). Prevalence among Barcelona residents was age-standardised (direct method) using the age-distribution of the Caritas sample. These analyses were stratified by type of Caritas’s service (DAS or HMS) and sex.

Also, multivariate logistic regression models were fitted to determine the association of poor mental health with the explanatory variables using the adult sample. Four dimension-specific models were fit, one for each housing dimension, where all of the explanatory variables of each dimension were entered into the model (models 1–4), and a final full model was fit including only the significant variables (model 5). Potential confounding by socio-demographic characteristics was adjusted for in all 5 models. These models were adjusted for men and women, and for DAS and HMS populations.

All analyses were conducted using Stata, version 11.2 (StataCorp LP, College Station, Texas).

## Results

### Description of socio-demographic and housing characteristics

Of the 320 total participants, 64.4 % were women. Table 1 displays socio-demographic characteristics of the sample stratified by Caritas’s service and sex. The vast majority (89 %) of the study population fell into the two most deprived social class levels (these social classes represent 35 % among Barcelona residents). Nearly one out of three (29 %) respondents had primary education or less (7 % in Barcelona). Foreign-born individuals made up a large proportion of both the DAS (93.7 %) and the HMS (57.9 %), the majority of which came from Central and South America. However, the legal situation of the immigrants differed between the two groups: 43.7 % of the DAS participants were undocumented immigrants compared to 2.1 % of the HMS sample. In regards to employment status, unemployment levels were high universally (53.1 %), with higher levels among men and the HMS, and half of those employed were working without an employment contract. Additionally, 70 % of households included one or more children, with women and those from DAS more likely to be single parents.

Table 2 shows housing characteristics. Regarding housing affordability, 87.6 % of participants spent more than 30 % of their monthly income on housing expenses, 86.8 % reported having trouble meeting their monthly living expenses, and 78.7 % had been worried during the previous year about not being able to afford food. With respect to housing instability, 53.1 % of HMS and 36.6 % of DAS participants had spent less than two years in their current home, and one out of five (21.3 %) had at one point lived in a shelter, shack, car, or on the street. With regard to satisfaction with the dwelling, one out of three (29.4 %) participants reported being unsatisfied or very unsatisfied with his/her dwelling. Concerning housing tenure, most HMS participants were homeowners (39.3 %) or renters (42.8 %), while the most frequent housing type among DAS participants were rented housing (45.1 %) or a rented room (37.7 %). The number of habitability problems as well as the number of housing-related material deprivations was higher among DAS participants. One out of five (22.1 %) of HMS participants and more than half (56.7 %) of DAS participants lived in an overcrowded dwelling. Finally, regarding neighbourhood conditions, one out of five (19.4 %) participants evaluated their neighbourhood negatively.

### Description of health outcomes and comparison with Barcelona residents

Table 3 shows the prevalence of health outcomes among the study population and Barcelona residents. Overall, health status of the Caritas population was much worse than that of Barcelona residents, even than that of most deprived social classes, both among adults and among children. Most health indicators were worse among women and among HMS participants in adults and among DAS participants among children. For example: 69.4 % of adult participants had poor mental health (11.5 and 15.2 % among men and women Barcelona residents). In children, prevalence of poor mental health among boys was of 61.3 % for those from DAS and of 45.2 % for those from HMS (3.1 % in Barcelona), and of 37.5 and 25.0 %, respectively, among girls (5.1 % in Barcelona).

**Table 3:** Prevalence (%) of health outcomes among the population assisted by Caritas Barcelona and comparison with Barcelona residents

	Men/Boys				Women/Girls			
	Caritas population		Barcelona residents		Caritas population		Barcelona residents	
	Direct Assistance Services	Housing Mediation Service	Overall	Deprived social classes (IV and V)	Direct Assistance Services	Housing Mediation Service	Overall	Deprived social classes (IV and V)
Adults								
Regular or poor self-reported health	24.4	45.8	11.2	12.8	53.1	60.3	15.3	20.5
Quality of life, below the median score in Cáritas	35.6	51.4	5.6	4.4	47.7	58.9	8.3	13.1
Poor mental health (Goldberg-12)	62.2	75.0	11.5	12.1	66.9	72.6	15.2	17.4
Self-reported depression or anxiety the previous year	57.8	70.8	5.8	6.8	63.8	86.1	9.2	10.6
Consumption of tranquilizers, antidepressants, or sleeping pills the previous 2 days	6.7	18.1	6.7	8.3	15.4	30.1	15.7	13.8
Self-reported migraine or frequent headaches during the previous year	28.9	38.9	3.6	3.6	62.3	65.8	8.5	7.9
Self-reported backache during the previous year	37.8	63.4	12.0	15.8	73.9	83.6	19.2	18.9
6 or less daily mean hours of sleep (working days)	45.5	56.9	22.1	22.1	53.1	61.1	24.9	30.6
Children								
Regular or poor health status	22.7	7.1	0.9	NE	22.9	22.0	1.7	NE
Poor mental health (SDQ questionnaire)	61.3	45.2	3.1	NE	37.5	25.0	5.1	NE
Recurrent otitis during the previous year	20.5	19.0	3.6	NE	31.9	19.5	1.6	NE
Not having had breakfast everyday before leaving home	15.9	26.2	6.4	15.4	20.8	22.0	8.4	14.5

*NE* Not estimated (due to small numbers)

### Association between housing and mental health

The multivariate association between housing and poor mental health is shown in Table 4. In men, in the final model (model 5) having been worried about not being able to buy food (OR = 14.2), having lived in a shelter, shack, car, or on the street (OR = 11.2), not being satisfied with the dwelling (OR = 6.6 and OR = 34.2 for the middle and lowest emotional link categories compared to the highest, respectively) were significantly associated with poor mental health, whereas living in an overcrowded home was inversely associated with poor mental health (OR = 0.2). In women, both in the dimension-specific and the full models, having been worried about not being able to buy food (OR = 3.2), having lived in a shelter, shack, car, or on the street (OR = 5.9), and living in a neighbourhood where noise impedes sleep (OR = 2.2) were associated with poor mental health.

**Table 4:** Association (OR [95 % CI]) between housing-related exposures and poor mental health among the population assisted by Caritas Barcelona Multivariate dimension-specific models (1–4) and full model (5)

	Men^a^		Women^b^		Direct Assistance Service^c^		Housing Mediation Service^d^	
	Models 1–4	Model 5	Models 1–4	Model 5	Models 1–4	Model 5	Models 1–4	Model 5
	Model 1		Model 1		Model 1		Model 1	
**Affordability**								
Problems meetingmonthly costs								
Strongly agree	1		1		1	1	1	
Agree	0.7 (0.2; 1.8)		1.0 (0.4; 2.3)		0.5 (0.2; 1.2)	0.5 (0.2; 1.0)^*^	1.7 (0.6; 4.7)	
Strongly disagree, disagree, indifferent	0.4 (0.1; 2.0)		0.7(0.3;2.0)		0.2 (0.1; 0.6)^***^	0.1 (0.0; 0.4)^***^	4.9 (0.9; 26.7)^*^	
In the last 12 months, worried about not having sufficient money for food								
No	1	1	1	1	1		1	1
Yes	8.8 (2.8; 27.7)^***^	14.2 (3.7; 54.9)^***^	3.3 (1.5; 7.5)^***^	3.2 (1.5; 7.0)^***^	2.7 (1.1; 6.7)^**^		8.9(3.2; 25.2)^***^	6.0 (2.5; 14.6)^***^
Time in current house								
≥ 2 years	1		1		1		1	
< 2 years	0.5 (0.2; 1.5)		0.8 (0.4; 1.7)		0.5 (0.3; 1.2)		1.3 (0.5; 3.6)	
Has ever lived in a shack, shelter, car, street								
No	1	1	1	1	1	1	1	1
Yes	7.5 (1.7; 32.9)^***^	11.2 (2.3; 55.2)^***^	5.7 (1.5; 21.4)^***^	5.9 (1.6; 21.8)^***^	4.8 (1.6;14.4)^***^	4.0 (1.4; 11.8)^**^	5.1 (1.3; 20.3)^**^	5.4 (1.4; 20.4)^***^
	Model 2		Model 2		Model 2		Model 2	
**Emotional link with the dwelling**								
Very satisfied or satisfied	1	1	1		1		1	
Indifferent	3.4 (1.2; 9.5)^**^	6.6 (1.7; 23.2)^***^	1.6 (0.8; 3.5)		2.0 (0.9; 4.4)^*^		1.9 (0.8; 4.8)	
Very unsatisfied or unsatisfied	12.5 (3.2; 48.4)^***^	34.2 (5.8; 201.9)^***^	2.1 (0.9; 4.7)^*^		4.8 (2.0;11.5)^***^		3.3 (1.2; 9.0)^**^	
	Model 3		Model 3		Model 3		Model 3	
**Dwelling Conditions**								
Housing type								
Apartment/single family home (rented)	1		1		1		1	
Apartment/single family home (owned)	0.6 (0.2; 2.4)		0.9 (0.3; 2.8)		0.4 (0.0; 6.0)		1.2 (0.4; 3.2)	
Room (part of a house)	1.1 (0.3; 4.3)		1.2 (0.4; 3.5)		1.1 (0.4; 2.9)		4.3 (0.3; 58.5)	
Other^e^	0.9 (0.2; 4.9)		0.8 (0.3; 2.1)		0.7 (0.3; 2.0)		3.1 (0.6; 14.6)	
Number of habitability problems (tertiles)								
0–1	1		1		1		1	
2	1.7 (0.6; 5.0)		2.2 (0.9; 5.3)^*^		2.4 (1.0; 5.8)^*^		2.6 (1.0; 7.1)^*^	
3–5	3.8 (0.9; 16.2)*		1.3 (0.5; 3.1)		1.8 (0.8; 4.1)		5.3 (1.1; 26.4)^**^	
Missing values	2.2 (0.3; 14.4)		1.3 (0.3; 5.9)		4.5 (0.9; 23.2)^*^		0.3 (0.0; 2.5)	
N°housing-related material deprivations (tertiles)								
0–3	1		1		1		1	
4	4.7 (1.0; 21.7)^*^		1.6 (0.7; 3.7)		3.2 (1.2; 8.4)^**^		2.4 (0.8; 7.3)	
5	2.0 (0.7; 6.1)		2.1 (0.9; 5.9)^*^		2.2 (1.0; 4.9)^*^		2.2 (0.6; 7.8)	
Overcrowding								
≤ 1 ppr	1	1	1		1		1	
> 1 ppr	0.4 (0.1; 1.2)^*^	0.2 (0.0; 0.6)^***^	1.3 (0.5; 3.2)		0.9 (0.4; 2.2)		1.1 (0.3; 3.3)	
	Model 4		Model 4		Model 4		Model 4	
**Neighbourhood and community conditions**								
Evaluation of neighbourhood as a place to live								
Very good, good or indifferent	1		1		1		1	
Very bad or bad	3.2 (0.8; 13.6)		1.5 (0.5;4.0)		2.2 (0.8; 6.1)		2.3 (0.7; 8.0)	
Noise								
Noise does not impede sleep	1		1	1	1	1	1	
Noise does impede sleep	1.4 0.6; 3.5)		2.4 (1.2; 4.9)^**^	2.2 (1.1; 4.4)^**^	3.6 (1.7; 7.7)^***^	3.7 (1.6; 8.2)^***^	1.0 (0.5; 2.2)	
Safe neighbourhood (when returning home at night) where violence is not a problem								
Strongly agree or agree	1		1		1		1	
Strongly disagree or disagree or indifferent	1.2 (0.5; 3.0)		1.5 (0.7; 3.2)		1.2 (0.6; 2.4)		1.6 (0.6; 3.9)	

*OR* Odds Ratio; *95 % CI* 95 % Confidence Intervals; *ppr* Persons per room

*p-value ≤0.10; **p-value ≤ 0.05; ***p-value ≤ 0.01

^a^All models are adjusted for age and Caritas’s service (DAS or HMS); ^b^All models are adjusted for age, Caritas’s service (DAS or HMS), and social support; ^c^All models are adjusted for age and sex; ^d^All models are adjusted for age and sex; ^e^Guesthouse, bed in shared bedroom, shelter, friend’s or family’s house, among others

Among DAS participants, in the full-model (model 5), having lived in a shelter, shack, car, or on the street (OR = 4.0), and living in a neighbourhood where noise impedes sleep (OR = 3.7) were associated with poor mental health, whereas not having problems meeting monthly housing costs was inversely associated (OR = 0.1). Finally, among those from the HMS, in the final model, only having been worried about having enough money for food (OR = 6.0) and having lived in a shelter, shack, car, or on the street (OR = 5.4) were significantly associated with poor mental health.

## Discussion

While general population surveys have largely failed so far to identify the negative health consequences of the current economic crisis, this study highlights that in Barcelona, people seeking help from Caritas and facing serious housing problems have a much worse health status than the general population, even when compared to those belonging to the most deprived social classes. This is especially so for users of the HMS, who specifically seek help for dealing with unbearable housing payments. Moreover, even within a small sample of subjects, all of whom are facing some kind of housing problem, housing conditions are related to poor mental health, even after controlling for other adverse life conditions such as unemployment or being undocumented. These findings are consistent with the growing body of literature showing the association between poor housing conditions, mainly housing affordability, and poor physical and mental health [4, 5, 15, 26, 27].

It is worth mentioning that the population assisted by Caritas has a high vulnerability profile, shown by their socio-demographic and housing characteristics as well as by their health outcomes. Difficulties in affording housing costs among this population is most likely due to the fact that the majority of interviewees are unemployed, mainly among men who tend to be the breadwinners. In addition, more than one third of women are single mothers, who have to face with both the responsibility of house and children care as well as with being in a precarious situation, as other studies have shown [28, 29]. Nonetheless, it is also worth highlighting that the sample was not only made up of the more excluded populations, but also included other groups that do not tend to use Caritas’s services, such as those from less-deprived socials classes.

Within the two Caritas groups, women had worse self-reported health, slept less, were more depressed or anxious, used medications more frequently, and had worse quality of life, which has been described elsewhere [28, 30, 31]. Additionally, users of the HMS had considerably worse health than those from the social services living in substandard housing. HMS users are mostly families approaching Caritas for the first time searching for help to deal with a mortgage or rental payment problem. Such struggles have been shown to increase psychosocial stress and anxiety, and homeowners frequently view these difficulties as a personal failure [4, 8, 32].

Our study found, across all groups, that those with affordability problems reported worse mental health, mainly among those who worried about not having sufficient money for food or those who had at some point slept in a shed, shelter, car, or on the street. The poor mental health outcomes suffered by this population, especially by the HMS group, are consistent with a number of studies that demonstrate the poor health outcomes of people who experience homelessness [33] or foreclosure [9, 34]. Additionally, across almost all groups, housing satisfaction was associated with mental health such that those with lower satisfaction reported worse mental health. However, we did not detect strong associations between poor mental health and dwelling or neighbourhood conditions. This might be attributable to the lack of variability in housing and neighbourhood characteristics among the study population, since the majority of study participants experience serious substandard living circumstances, therefore making it difficult to tease out associations with health outcomes in this relatively small population.

In men we found that overcrowding was associated with better mental health. This was surprising given that some studies indicate that overcrowding can result in worse physical and mental health outcomes due to higher rates of infectious transmission and other poor living conditions suffered by those living in overcrowded homes and the stress associated with being unable to pay one’s bills which could force one to move in with others [35, 36]. However, the fact that these men live in an overcrowded home could indicate that they have a social safety network to fall back on in difficult times. Such social support could lead to improved mental health. Future studies should confirm this hypothesis. This association was not observed among women, which could be explained by the social construction of gender [24]. The role of breadwinner, assigned to men, represents a source of pressure in times of economic difficulties. Similarly, the association with the emotional link with the dwelling was observed only among men, since the male figure is responsible for providing his family with an adequate housing.

The study had a few limitations. Our sample size was relatively small due to the difficulties in reaching these populations representing multiple types of housing instability, both those who live in substandard housing and those who struggle to pay housing costs. However, our sample was large enough to analyse the life conditions and health status of a very difficult to reach population, often underrepresented in general health surveys. Perseveration and close follow up of individuals was needed, for which the implication of Caritas in the study was essential. The economic and legal instability of these families, their proneness to mobility, and their precarious situation were important barriers to participation in the study. Incentives were not used in this study. Differences between participants and non-respondents could not be analysed, although most likely non-respondents were those in more precarious situation and, therefore, most likely to have worse health status. Additionally, the cross-sectional nature of the baseline survey prohibits us from drawing causal inferences from the findings, although cohort studies done in other countries have found similar associations [32, 34]. Also, all participants were drawn from the Caritas registry and as such may not accurately represent all of those living in substandard housing in Barcelona, only those who are seeking assistance. Finally, participants were younger than overall Barcelona residents and mostly foreign-born. Since age is related to prevalence of most health problems, age-standardized prevalence was used instead. Among the strengths, participants responded to three thorough questionnaires, with most of the items consisting in validated instruments. Additionally, this study is the first to compare the health outcomes of a vulnerable population with housing instability with those from the overall population of Barcelona, and therefore contributes to the scarce literature analysing the impact of housing on health in Spain, a country characterised by having a housing system with limited access to adequate housing.

## Conclusion

This study has shown that, in a country hit by the financial recession, those people facing housing problems have much worse health compared to the general population. Housing affordability should be a priority for policymakers, and urgent action is needed to increase the supply of public social housing and to guarantee people’s right to adequate housing, including measures to stop foreclosures or to implement deeds of assignment for mortgage payments.
